# Designing front-of-pack labels for packaged food in low-literacy contexts: Consumer perspectives from India

**DOI:** 10.1371/journal.pgph.0007118

**Published:** 2026-08-25

**Authors:** Lakshmi K. Josyula, Simone Pettigrew, Palak Mahajan, Claire Johnson, Devarsetty Praveen, Rachita Gupta

**Affiliations:** 1 The George Institute for Global Health, Hyderabad, India; 2 The George Institute for Global Health, Sydney, Australia; 3 University of New South Wales, Sydney, Australia; 4 Prasanna School of Public Health, Manipal Academy of Higher Education, Manipal, India; 5 World Health Organization Country Office for India, New Delhi, India; PLOS: Public Library of Science, UNITED STATES OF AMERICA

## Abstract

Front-of-pack labels (FoPLs) on food products are an evidenced-based, effective strategy to raise consumer awareness of the composition and (un)healthiness of packaged foods and help consumers make informed food choices. We undertook a community-based study, in two regions in India, to understand food purchasing patterns and the essential content and design of an effective and contextually acceptable FoPL, and to gather consumer input on a draft FoPL developed by the Food Safety and Standards Authority of India. We conducted 16 focus group discussions with 112 adults, who were regular grocery shoppers for their families. Participants were selected purposively for diversity in gender, educational attainment, socioeconomic status (SES), and site of residence. Data were analysed thematically. Nutrition literacy was revealed to be quite low in most participants, particularly those with low educational attainment, and of low SES. Purchases of packaged foods, including snacks, beverages, and ready-to-eat meal components, were reported to be guided by habit, trust, taste, and others’ recommendations. Nutrition information influenced food purchasing decisions mostly for those following diets for various health conditions. Nutrition information on products, although desired, was not always understood correctly. Symbols without supporting text were sometimes interpreted incorrectly. Interpretive FoPLs with clear, readable text, and colours were preferred, and almost all participants wanted FoPLs to be mandatory. A robust nutrition literacy campaign is essential to set the stage for, and accompany the effective implementation of, an FoPL. An ideal FoPL for India would be mandatory, provide nutrition facts, and also interpret them in a summary format for consumers’ benefit. Information in languages suitable for the diverse population combined with colours and symbols would enhance the utility of the FoPL.

## 1. Introduction

Front-of-pack labels (FoPLs) on food products are an established, evidence-based, effective strategy to raise consumer awareness of the composition and (un)healthiness of packaged foods and help them make informed food choices [1–5]. The Food Safety and Standards Authority of India (FSSAI) is in the process of developing a national FoPL tailored to the population’s needs, guided by the experiences of numerous countries across the world, and supported by the protocols established by global nutrition expert bodies [6]. Codex and World Health Organization (WHO) FoPL guidelines have emphasised the need for FoPLs that will increase consumer understanding of the healthiness of food products, be acceptable and facile to use, and promote reformulation of products as called for [7,8].

India has a highly diverse population in terms of educational attainment, nutrition literacy levels, socioeconomic status, geographic location, residential arrangements, traditional cuisines and modern food markets selling processed packaged foods, epidemiological transition, social and cultural practices, and access to health and nutrition information. This diversity is likely to present challenges for designing and implementing an effective FoPL applicable across the country. It is therefore essential to gain a nuanced understanding of consumer knowledge, attitudes, preferences, and practices relating to FoPLs, and the gaps that need to be addressed to facilitate the introduction of an effective FoPL in India. The array of foods in the market has increased considerably in recent times [9,10], adding to consumers’ difficulty in successfully evaluating the (un)healthiness of various food products and making choices. An effective FoPL system needs to communicate complex information to consumers in an easily understood and standardised format to shape healthy food choices and purchasing behaviour.

A review that we conducted of the research and policy literature on FoPLs revealed a scarcity of studies examining consumers’ understanding and the impact of FoPLs on their purchasing choices. The evidence on buying decisions is drawn from simulations and modelling, and laboratory settings [11] rather than real-world observations, particularly in the South and South-East Asian regions [12].

FoPLs can be broadly classified as reductive or interpretive. Reductive labels provide nutrition facts, and interpretive labels provide indications of the (un)healthiness of the food product or its nutritional components [13]. Interpretive FoPL formats are considered easier to understand than non-interpretive formats, holding the potential to promote healthier food choices [14], although relative performance of different interpretive FoPLs is not consistent [15]. Literacy, in particular health and nutrition literacy, is important to the successful use of FoPLs, particularly reductive FoPLs that present nutritional information sans interpretation. Comprehensibility of FoPLs is helped by a viewer-friendly large font size, colours on the label, and contrast between the FoPL and the background [16]. Symbols have been shown to have the potential to perform better than text, as long as they are not ambiguous or difficult to interpret [17]. The paucity of evidence highlights the need for region-specific understanding through studies in real-life settings in different populations before FoPL implementation [4,18].

The aim of the present study was to understand packaged food purchasing patterns and decision-making related to packaged food purchases, obtain consumer input to enhance the utility of a draft FoPL developed by the FSSAI, and to understand the essentials of content and design of an FoPL for effectively communicating the unhealthiness of certain foods, promoting healthier alternatives, and influencing purchase behaviours.

## 2. Materials and methods

This study was a part of a larger project examining a range of FoPLs that would be most appropriate for the Indian population [19]. In the present study, 16 focus group discussions (FGDs) were conducted with 112 persons, 58 women and 54 men, in two regions (Haryana and neighbouring areas in the North, and Telangana in the South) in India, between May and October 2021, to understand features of FoPLs that would be most useful for, attractive to, and desired by Indian adults; and to arrive at the elements of an FoPL suitable for Indian consumers, in particular, effective in enhancing consumer awareness and understanding. Participants were adult community members who were regular grocery shoppers, purchasing packaged foods, such as snacks, beverages, and ready-to-eat meal components, for their households. They were selected purposively through the peer and professional networks of the researchers, including NGO collaborators and community representatives who had participated in other field studies conducted by the researchers. Participants were brought together in internally homogenous groups that differed from one another in sociodemographic characteristics such as gender, SES, educational attainment, and site of residence. The FGDs were conducted in different regions (Delhi, Faridabad, and Ghaziabad in North India, and Siddipet and Hyderabad in South India), and locations (urban, urban slums, and rural) to achieve variation in participants’ regions and conditions of residence and dietary patterns, and also variation in food baskets accessed by people. Approaching participants from urban, urban slum, and rural settings facilitated selection of participants of diverse levels of educational attainment, occupations, and household incomes. These distinct geographic zones were also a pragmatic selection from the standpoint of the research team’s familiarity with the regions, and access to field staff through the institution’s offices and project sites in these zones.

**Fig 1:**
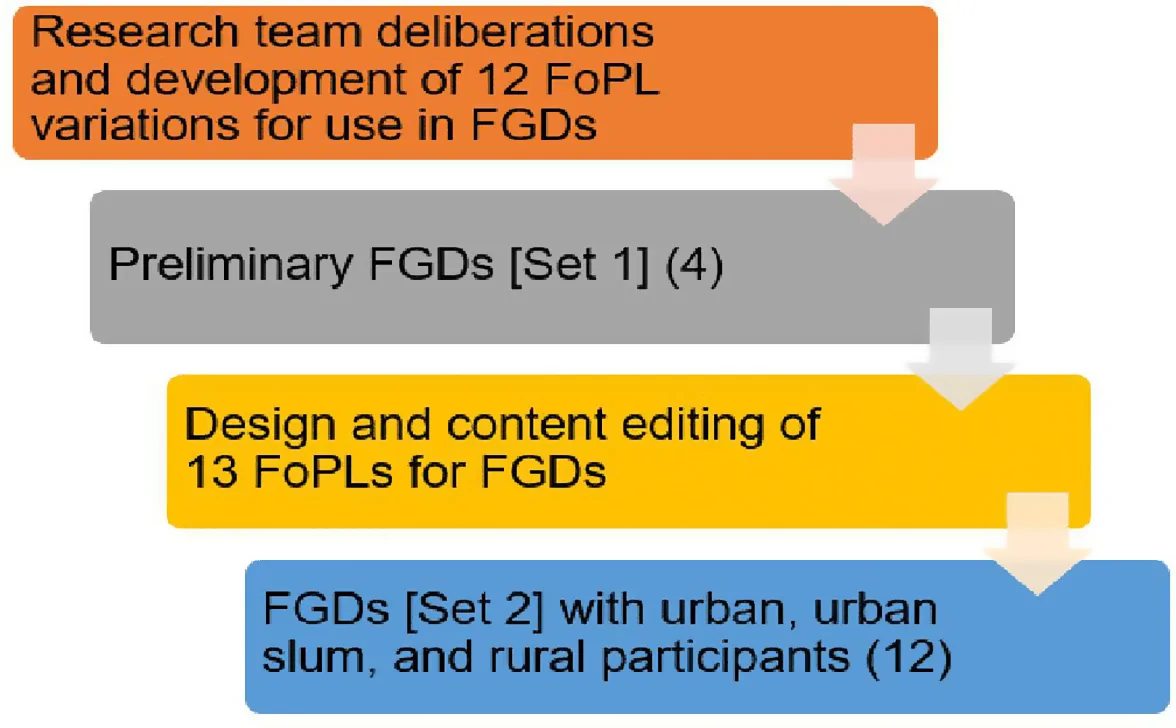
Sequence of FGDs and FoPL iterations.

Our study was iterative: We conducted the preliminary FGDs, and informed by our findings made several revisions and modifications to the FoPLs that we used for the subsequent FGDs. Research team discussions while data collection was ongoing informed modifications to the FoPLs used in the FGDs and the topics that we delved into in the discussions as well. The FGDs were conducted in two sets (see Fig 1): First, a preliminary set of four FGDs was conducted to gather feedback on the content and presentation of FoPLs that would be most useful for consumers, and on the FoPL variations that we had developed. Several revisions were made to the FoPLs based on the feedback from these preliminary FGDs. Second, a subsequent set of 12 FGDs was conducted using the revised FoPLs, to explore patterns of food purchasing, consumers’ assessment of the healthiness of foods, challenges encountered in evaluating the (un)healthiness of packaged foods, knowledge of current on-pack labelling and nutritional information, and information desired on food labels (S1 File). The FGD guide was developed specifically for this project in view of the paucity of prior comparable research conducted in LMICs. The topics in the FGD guide, as well as the choice of FoPL variations used in the FGDs, were based on our review of the relevant literature and reports of food consumption patterns and trends in India [20,21]. Participants discussed the revised FoPLs displayed, and ranked them on attractiveness and usefulness at the conclusion of the FGD. Details of the FGDs and participants are presented in Table 1.

**Table 1:** Focus group discussions and participant profiles.

Discussion	#	Site/ Zone	Participants (number/ gender)	Socio-economic status	Education	Language
Preliminary focus groups [Set 1]	1	Urban	4 (1/F, 3/M)	High	High	English
	2	Urban	9 (5/F, 4/M)	High	High	English
	3	Urban slum	7 (5/F, 2/M)	Low	Low/none	Telugu
	4	Urban	6 (2/F, 4/M)	Med	Med	Hindi
			13/F, 13/M			
Focus groups [Set 2]	1	Urban/N	9/M	Low	Low	Hindi
	2	Urban/S	4/M	High	High	English
	3	Rural/N	9/M	Low-med	Low	Hindi
	4	Rural/S	6/M	Mixed	Med	Telugu
	5	Urban slum/N	7/M	Low-med	Low-med	Hindi
	6	Urban slum/S	6/M	Low-med	Low-med	Telugu
			41/M			
	7	Urban/N	7/F	High	High	English
	8	Urban/S	5/F	High	High	English
	9	Rural/N	7/F	Med-high	Med	Hindi
	10	Rural/S	6/F	Mixed	Med	Telugu
	11	Urban slum/N	7/F	Low	Low	Hindi
	12	Urban slum/S	13/F	Low	Low-med	Telugu
			45/F			
			112 (58/F, 54/M)			

N = North; S = South; med = medium.

Education: (i) High = post-graduate/ professional; (ii) Med = secondary to high school; (iii) Low = no schooling to primary school.

Based on assessments of the nutrient profiles of products available in the Indian marketplace [20], we selected the following food products (commonly consumed across socioeconomic strata in India) for presentation in the FGDs: biscuits, roasted chana (Bengal gram), roasted peanuts, fried peanuts, namkeens (savoury fried snacks), buttermilk, cola, potato chips, and instant noodles. These foods were purposively picked to result in a mix of mostly healthy and mostly unhealthy products to assess the accuracy of comprehension of FoPLs. We undertook an iterative process of design development of FoPLs to be placed on mock food packages for use in the FGDs. A graphic designer developed the images of the mock packages using stock images of foods, and fictitious names were assigned to the products. The research team made five language, colour, text, and image variations of the draft FSSAI FoPL notified by FSSAI in 2022, a hybrid model which had % RDA contributions, and highlighted nutrients higher than the recommended threshold in red colouring to warn consumers (S2 File). In addition, we selected the following six popular FoPL formats in use in various parts of the world: Multiple Traffic Lights (MTL), Guideline Daily Amount (GDA), Health Star Rating (HSR), Nutri-Score, Israeli Warning Label, and Chilean Warning Label. These eleven FoPLs, which varied in terms of the design, nutritional components presented, units of measure used, indicative colours to aid interpretation, and language, were then applied to a mock food package each.

Members of the research team, who were trained and experienced in conducting interviews and FGDs, collaboratively finalised the FGD guide in the planning stage. They were then observers at the preliminary FGDs (Set 1) in various languages, moderated by senior researchers of the team. All team members participated in research team debriefing sessions after each preliminary FGD. Following this, three members of the team, singly or in twos, as called for by the language and location requirements, moderated the FGDs in Set 2 (see Table 1) applying an approach consistent with that used in Set 1. Each FGD began with a discussion of food purchasing practices and preferences. We then described the mock food packages and broadly what an FoPL was expected to do, i.e., provide a capsule of information about the package contents. Participants in the four preliminary FGDs (see Table 1) provided feedback on the content and design of the FoPLs. They recommended larger font sizes and helpful images for clarity. They welcomed the use of colours as an interpretive aid (red indicating unhealthiness, and green indicating healthiness), as well as the provision of information in multiple languages to be accessible to more consumers. Urban professional participants recommended standardised content, such as displaying nutrient composition per 100 g or 100 ml rather than per serve (which could be variable across food products, and confusing). Several terms used in the study FoPLs, and some images too, were interpreted variably by participants. For instance, ‘energy’ was interpreted as a positive thing by persons of lower socio-economic status (SES), whereas ‘calories’ was considered something to limit. The image of drops indicating fat was interpreted as water by some participants and as eggs by some others.

Following the preliminary FGDs, the study FoPLs underwent several revisions based on feedback from the participants. To ensure that participant feedback was systematically translated into revisions of the draft FoPLs, an iterative, team-based analytic approach was utilised. After each of the preliminary FGDs, members of the research team conducted structured debriefing sessions in which key observations relating to comprehension, interpretability, visual salience, and perceived usefulness of FoPL elements were documented. We also obtained input on our findings from the FGDs, from members of the Technical Advisory Group that we had constituted for this project. These inputs and our ongoing review of the literature on FoPL content and design informed our revisions and refinement of the FoPLs that we used in further data collection.

Changes that were supported by recurring participant feedback and judged likely to improve clarity and interpretability were prioritised for incorporation into revised FoPL designs. For example, the replacement of the term ‘sodium’ with ‘salt’ was informed by repeated evidence of misunderstanding of technical terminology, particularly among participants with lower nutrition literacy, and by participant preference for commonly used, non-technical language. Similarly, revisions to colour schemes, symbols, and the shift from ‘per serve’ to ‘per 100 g’ presentation were based on observed difficulties in interpretation and comparability across products. The word ‘sodium’ in the FoPLs was changed to ’salt’ in all FoPLs except the HSR. The ‘per serve’ value on all FoPLs, except one draft FSSAI version, was changed to ‘per 100 g’. One FoPL retained ‘per serve 30 g’. We retained some features unrevised on at least one FoPL, as detailed above, to assess the acceptability and utility of the original format in other focus groups. The text on one FoPL was converted to symbols: a thunderbolt icon for energy, an oil can icon for saturated fat, a sugar cube icon for sugar, and a salt-shaker icon for salt. The symbols were revised to make them more contextually relevant and better understood. All the FoPLs that had five nutritional components displayed were modified to display four components (energy, saturated fat, sugar, and salt) in line with the FSSAI recommendations, which were updated during the course of the conduct of our study. An additional draft FSSAI label variation was made with the nutrient information colouring moved to outside the crescent area. The FSSAI FoPL variations were coloured (red and/or green) using the WHO SEARO nutrient profiling model [21]. Five out of the six variations used only red colouring while one variation used both red and green colours. Any nutrient above threshold level, as defined for the specific food category, was marked in red, while any nutrient below the threshold level was marked in green. The use of three colours in the FSSAI variations was avoided to ensure distinction from the MTL FoPL. The non-FSSAI FoPLs (HSR, MTL, Nutri-Score, Warning Labels, and GDA) were coloured and/or marked high/med/low according to the relevant algorithm cut-offs for each FoPL (WHO-SEARO cut-offs were not used in designing these six FoPLs for the FGDs). Participants in the second set of 12 FGDs were shown FoPLs with the revisions (S3 File) on images of packs of roasted chana, roasted peanuts, fried peanuts, potato chips, namkeen mixture, biscuits, instant noodles, buttermilk, and cola.

Of the 12 FGDs, which followed the four preliminary mixed-gender FGDs, six were conducted with men and six with women, in the two study zones (see Table 1). Ten to fifteen participants were invited for participation in each FGD, and an average of seven persons participated in each FGD. At the conclusion of each FGD, participants were asked to vote for the top three FoPLs out of the 13 shown to them that they judged to be most useful as aids to food purchasing decisions. This process took the form of reviewing all the FoPLs, and eliminating, through on-the-spot calling out of votes for or against, the ones that the discussants did not find particularly attractive and useful. Then, the shortlisted FoPLs, five to six in each group, were displayed and evaluated again and ranked. All the FGDs were conducted in English, or the local languages, Telugu and Hindi.

The FGDs lasted 30–70 minutes each, with an average of 45 minutes. FGDs were moderated by one or two members of the research team, and conducted in English, Hindi, Telugu, or a combination of these languages as called for. The research team had debriefing sessions following every two or three FGDs, to discuss preliminary findings, participants’ feedback on the FoPLs presented, and topics to delve into in subsequent FGDs. All FGDs were audio-recorded and transcribed in English. Transcription was undertaken by members of the research team, who were proficient in the local languages, after translation from Hindi and Telugu if required. Transcripts were verified against audio-recordings by the moderators, read by the research team, and discussed for clarity and development of major themes at the research team meetings. They were then interpreted and synthesized thematically, in alignment with the themes in the FGD guide and other themes that emerged from the data. No qualitative analysis software was used for this analysis. Analysis was led by the first author, and conducted in close coordination with the third author, of whom one or both were present at every FGD conducted. Codes (e.g., ‘trust in brand’, ‘price’) and subthemes (e.g., ‘perceptions of language on FoPLs’, ‘information perused on food packages’) under the major themes (e.g., ‘purchasing decisions’, ‘nutrition literacy’) were developed from the reading of the transcripts and discussions of observations at the FGDs. Research team deliberations facilitated the refining of codes, combining some and adding others, and were a forum for conflict resolution and consensus building on thematic analysis as well. Analysis began right after the preliminary FGDs and proceeded concurrently with data collection thereafter.

### 2.1 Ethics statement

Ethics approval was obtained from the institutional ethics committee of The George Institute for Global Health, India. All participants provided verbal informed consent, documented on paper forms by the research team members, prior to the FGDs. Five of the FGDs were conducted online (primarily to reduce the risk of COVID-19 infection) and eleven were conducted face-to-face following all precautionary (COVID-19) measures to ensure that participants in urban slum and rural areas were represented regardless of access to the Internet.

## 3. Results

**Fig 2:**
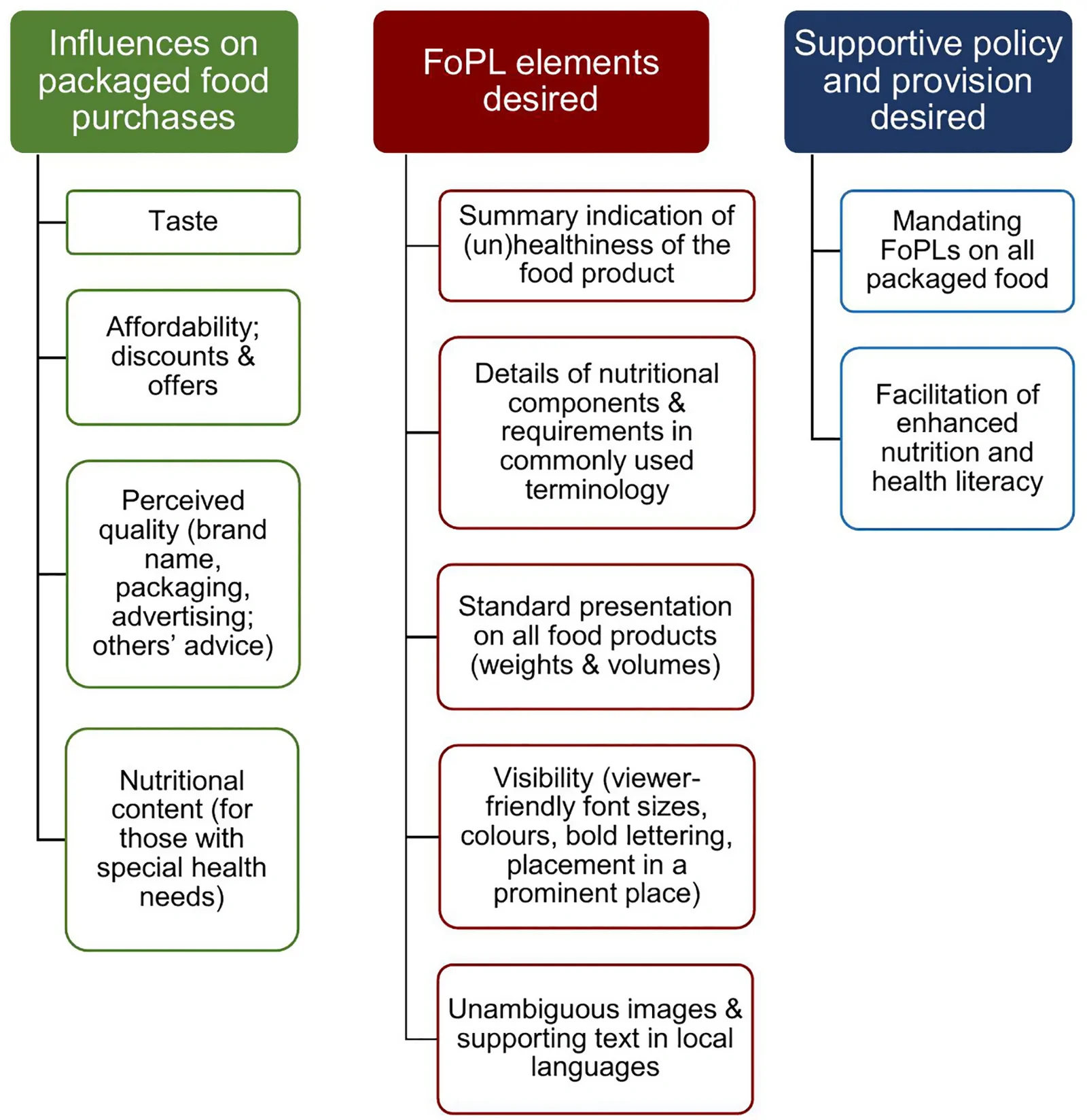
Purchasing decision influences, and perspectives on FoPLs.

The FGDs with adult participants from two different regions in India provided information on food purchase patterns, decision-making on food purchases, use of and desire for nutritional information and guidance on the healthiness (or lack thereof) of various food products on food packages. Participants provided detailed feedback on the design and content of the FoPLs presented in the FGDs, and also about other elements that they believed would be useful for large scale deployment of the FoPLs. The observations are encapsulated in Fig 2, and detailed below, with illustrative quotes.

### 3.1 Packaged food purchasing patterns

Packaged foods formed a regular and substantial proportion of the usual grocery purchases of medium-high income participants. Low-income participants reported that the main form of packaged products that they purchased were snacks, purchased mostly by and for children. Among adults, packaged snacks were consumed more often by men than women, particularly outside the home. Packaged snacks were purchased and brought into the homes of low-income participants mostly for celebratory gatherings. Some participants spoke of widespread adulteration of packaged food products that dissuaded them from buying most packaged foods.

*[FGD 5] Children eat such food [instant noodles and soft drinks] everyday… We don’t eat it everyday, only sometimes when we get the opportunity.*

*[FGD 1] It is like that necessary items we buy in loose… Packed items are there, but mostly items in loose are bought. Packed item is bought rarely. The type of work we do we cannot afford expensive products.*

*[FGD 11] Packaged food is not all right. If you heat it, it smells* [becomes unsafe]*. But if children see these foods, they keep asking for them. The best is home food, but where do children eat homemade food?*

*[FGD 3] I have seen how <brand A> juice is filled in packs named <brand B> and sold. If you see the conditions in which the juice is filled, you will not buy juice again.*

The frequency of grocery shopping varied by category of food purchased. Fresh produce was reported to be purchased on a weekly or twice-weekly basis, while dry goods, such as grains, legumes, spices, and oils were purchased monthly. In low-medium income groups, savoury snacks, soft drinks, and some sweet snacks were purchased daily, particularly for children, and by men for themselves outside the home.

### 3.2 Influences on purchasing decisions

Purchasing decisions for packaged foods were reported to be made on the basis of taste, brand name, branding, packaging, perceived freshness, advertising, discounts, and sales offers. Trust in the product was deemed crucial in regular purchases, but not quite as much in occasional purchases. For low-income participants, affordability was considered an additional factor influencing purchasing decisions. Low-income participants reported first checking the price, and then expiry date, to guide the purchase of their preferred packaged food products. Participants in a rural FGD observed that goods past their expiry date were sold in villages at a cheaper price to consumers who were unaware of the expiry date and not sufficiently conscious of the importance of food safety. Some participants cited attractive packaging as a factor encouraging the purchase of packaged food products. Some also mentioned taking recommendations from shopkeepers or friends before purchasing packaged food items.

*[FGD10] According to the kids’ taste, what we buy differs. If it is <brand C>, it is more of salted chips and if it is <brand D>, it is more of chillies.*

*[FGD 7] When <brand E> came I just got it because of the advertisement, not because I saw anything or tasted it. Just because of the advertisement, so I think it comes to brand value… the advertisement.*

*[FGD 5] There is a difference between what a company makes and what a local person makes. The local one, you’ll get more quantity for the same price, so people prefer that sometimes.*

*[FGD 9] We want a good-looking packet. It helps us choose among brands.*

*[FGD 2] I have never looked at a label and tried to figure out what is in the food. I rely on tribal knowledge. I hang around with friends a lot, and what they say is good is what I buy.*

### 3.3 Seeking and using nutrition information on packaged food

Information on the nutritional value of food products was not sought or understood by most low-medium income participants, particularly when making routine purchases of packaged food brands that they were used to and trusted. The few exceptions to these habitual, unreviewed purchases were purchases of foods that household members with diagnoses of hypertension or diabetes mellitus might consume, in which case, the salt and sugar content respectively were reviewed by some shoppers; and situations in which they considered switching brands. Some low- and middle-income participants reported browsing YouTube for guidance on particular foods.

For some participants, being on a particular diet entailed examination of nutritional components, and therefore close attention to the nutritional panels of packaged foods. Participants suggested that some, or a lot, of the consumption of packaged food in India was based on consumers’ lack of awareness of the health hazards – owing to the paucity of guidance on packs of food products, and people’s inability to read and comprehend the health information presented, if any. They cited instances of their literate children’s drawing their attention to information on food packs and their acting upon such information.

*[FGD 4] People do not see that much* [are not so attentive]. *But if they are under a doctor check-up, they will be careful about soft drinks. No one will check until a health issue arises… When people buy snacks, they don’t see all these things.*

*[FGD 7] In the month I was doing the diet, I had to look at the nutritional content of the particular packet to be able to measure, because I had to be on a particular ratio to maintain. When I am not on a diet, I just buy things that have a discount on them.*

*[FGD 3] If people were to read information on these foods before eating them, nobody would eat these foods!*

*[FGD 11] If one is literate, one can read this. Since we cannot read* [English]*, if something that we buy is expired, our children read and tell us, and we return the goods we purchased.*

In contrast to low-medium income participants, high-income groups reported usually evaluating the healthiness of food products before purchasing them. The easy availability of a wide range of fresh and dry goods online and in local markets, and the trends towards earlier development of several non-communicable diseases and overweight and obesity among urban professionals were reported as prompting some investigation into foods and diets. Many participants who were interested in the nutritional value of the food they consumed sought information from the internet. High-income participants with higher educational attainment sought information from educational sites and online reporting that they considered well-researched and credible. Many participants reported taking the unhealthiness of packaged foods, particularly deep-fried savoury snacks and high-fat desserts, for granted, and not expecting them to contribute positively to their health, and did not examine food packages for details of nutrient composition of the foods that they bought. In many of the groups, participants remarked that people seeking ‘junk food’ already knew that it was unhealthy, and would not delve into the nutritional information on the pack, whereas purchases of healthy foods, or foods expected to contribute to better health, would have consumers examining information on the packs for particular nutrients and overall healthiness.

Participants who sought information online to supplement or corroborate information on food packages opined that reliable sources of information on nutrition were difficult to identify, and that information available online was inconsistent and often contradictory. They considered that FoPLs, notwithstanding the limited information on them, would help in comparing products, and could prompt consumers to seek more information from the nutrient profile on the back of the pack.

*[FGD 2] If you have a symbol or a mnemonic to indicate that something is healthy or unhealthy, there is no need to give details of the components. People can look at the back of the pack.*

*[FGD 7] There are certain brands that have become very popular. Secondly it is the cost that comes in. You will look for the cost online and figure out which one you want to buy. And now I guess recently I have become conscious of health aspects of things, like I am trying to switch from rice to brown rice… when you’re trying to figure out those things.*

*[FGD 2] I would never look at calorie count, say, when buying dal or chana. I wouldn’t bother with it even if I were buying chips, which I know are unhealthy. The place where information on the pack is useful is when you are trying to buy healthy food and have to decide between comparable options.*

Nearly all the participants who sought or skimmed through on-pack nutritional information reported focussing narrowly on one or a few nutritional components or characteristics, such as protein, vitamins, minerals, fibre, or calories. Usually the main focus was on the component highlighted in the health claims made on the package, or in advertisements, by the manufacturer. Some participants related the surprising lessons that they learned about the nutritional contents and health effects of foods that the health claims on packaged food had misled them about. Some participants also alluded to the tendency of consumers to take information on packs for granted and not pay attention to it after getting accustomed to new packaging.

*[FGD 5] Information is provided on the back of the packs, and we don’t pay attention because we are in a hurry. We only see the expiry date… If this information is provided on the front of the pack, it will be attractive for a few days because it will be new. Then after a while, it will be the same thing.*

*[FGD 7] Foods that are termed as healthy, like you know, whether it be your atta bread or this whole wheat bread, or whether it be digestive biscuits… The other day I was reading that digestive biscuits have more calories probably than your chocolate chip biscuits, hello!*

The majority of the participants from low- and medium-income groups reported being familiar with ISI [Indian Standards Institute, a government organisation under the Bureau of Indian Standards] and Agmark [Agriculture Mark, a mark designed to assure the quality of agricultural products in India] certifications on packaged foods, and mentioned such indicators as what they looked for on packs of prepared food and other products that they considered purchasing. This finding was unexpected as processed packaged foods sold in India do not have all these certifications. It is possible that participants were extrapolating from their experiences with other products. Participants across income groups were aware of the presence of nutrition information on the back of packs, but very few reported examining such information either before or after a purchase.

### 3.4 Visibility and detail of FoPLs

Participants in all the FGDs generally found the FoPLs rather small and the font size difficult to read, requiring close examination in-person or magnification online. While the participants recognised that the space available on a food package was limited, they remarked on the low visibility of small FoPLs to consumers, the limitations on the detail that could be displayed on small FoPLs, and therefore the limited utility of small FoPLs in providing information and aiding decision-making. To offset the small size of the FoPLs, some participants recommended clear fonts, bold lettering, and sharp contrasting colours. They also recommended that the FoPL be placed in a prominent place on the pack, such as next to the brand name, to ensure that consumers do not miss the information on it.

### 3.5 Interpretation of information in FoPLs

Participants across groups remarked on the lack of clarity of the values of food components presented as absolute amounts (e.g., grams and milligrams) and as percentages. Some participants suggested that nutritional information should be presented in a standardised manner on all packaged food, by all manufacturers, for the ease of consumers. The information in the FoPLs presented was found to be quite confusing and variably interpreted by participants.

*[FGD 4] We see the label. We see the manufacturing date, that is compulsory. Vitamins and minerals will be written. We can check the energy level… Energy and fat also. Fat should be less. Basing on health if we see, the fat content should be less… Salt percent should also be less… Energy should be high… Sugar should be medium. If there is a lot of sugar, we may suffer health problems.*

*[FGD 10] Respondent 1: In this pack, the salt is very less. It is only 2.2, but the energy is 533. I feel if the salt is less, the energy would be less.*

*Respondent 2: No, if the salt is less, the energy levels will be high.*

*[FGD 7] We don’t understand the packages. If you have highlighted 9%, but not 14% and 10%, are you trying to say that saturated fat is better than salt? Or that you can exceed the permissible limit of saturated fat, but not salt? You should put an asterisk there and explain it somewhere on the packaging so I can understand.*

*[FGD 2] If information on a pack is relatable to the way people consume the food, it would be usable. But when it says per 30 g, and I am eating cornflakes, I don’t know what 30 g is. If the information were per bowl, I could make better sense of it. Like if you are giving information about oil, you could give it in teaspoons, so I will know what is in 3 teaspoons of oil that I use to fry an egg. 20 ml is very difficult – to pour into a pan.*

Participants mentioned using their prior experiences as a guide to interpretation of FoPL colours and symbols. For instance, young mothers in rural areas connected their experience of using colour-coded growth charts for their infants and children in Anganwadi centres (government-run nutrition and education centres for perinatal women and children) with the colour-coding on some of the FoPLs. Many participants understood the HSR as soon as they viewed it, based on prior experience with star-rating labels on electrical appliances.

*[FGD 10] In the Anganwadi, there are small children. We know from the child’s age, if the weight is less and if it comes under the red mark, then we can say their health is in danger… Yes, and if we see the green mark then it is okay for them, and if it is yellow then it is getting down. We can understand seeing that. Weight should not be in yellow, it should be in green.*

There appeared to be scope for confusion among literate participants with low levels of education. Certain terms were read incorrectly, and other terms were read correctly but interpreted incorrectly by some participants. For instance, some participants from low-medium income groups misread ‘quantity’ as ‘quality’; and interpreted ‘energy’ as a positive feature of packaged food, not equated with calories.

### 3.6 Language and accessibility of FoPLs

Participants welcomed information in as many languages as possible in order for the FoPLs to be understood by a wide audience. While they acknowledged that it would not be easy to feature information in all languages used in India on each pack, they felt that at least two languages, English and Hindi, were the minimum required for utility across the country, and that the regional language added would greatly enhance FoPL utility. An insight that some participants shared was that if information were provided only in the local language, consumers might not be very keen to buy the product as it would seem like a locally manufactured product rather than a national or international brand.

*[FGD 4] If they write only in Telugu, some people will feel that it is a local brand, and they won’t buy it.*

*[FGD 10] People who know English can read whatever is written in English. People who know Hindi also can see the mark and understand. If it is in Telugu, many people can easily understand and there won’t be any problem. Not all people are educated, some people have less education, and they can understand Telugu, but not English.*

*[FGD 5] It should be in Hindi. The majority of people can read Hindi. We can read a bit of English too, but not everyone can.*

Some participants pointed out that although they knew their language in general, technical terms were often beyond their comprehension. Terms in common use were preferred over technical terms for greater relevance in consumers’ lives. For instance, participants from high-income groups suggested that information be displayed for ‘salt’ rather than ‘sodium’. Back-of-pack information (i.e., the nutrition information panel) was reported to present several barriers to comprehension, including inability to read English language text, difficulty understanding the technical information in the panel, small text size, and use of complex terms related to nutrition. The utility of the nutrition panel was considered quite limited, and participants did not think that the mere shifting of such highly technical information to the FoPL would be beneficial to consumers.

*[FGD 9] Sometimes we can read what is written, but we don’t understand what it means, and what it is about. Sometimes things are written in short forms – if anyone at home understands them, we can ask them. But otherwise, it is confusing.*

*[FGD 8] These are a little cryptic… We can figure it out, but if they say ‘salt’* [rather than ‘sodium’], *it would be a little better.*

*[FGD 12] We don’t know the difference between normal fat and saturated fat.*

*[FGD 8] Respondent 1: Who could possibly understand this term?! Only someone who has studied biology in Hindi may understand it.*

*Respondent 2: It is a good thing that they have used another language, but if this is the language that is used, the whole purpose is lost. Only some Hindi pundits will understand it!*

Non-text communication on the FoPL was not uniformly understood as intended. Participants with higher levels of education and those who could read and understand the text on the FoPLs felt that the use of multiple colours aided comprehension. However, while all participants appreciated colourful FoPLs as visually attractive, particularly those featuring multiple colours, many did not interpret the colours as indicative of (un)healthiness. In the absence of explanatory and supporting text, non-text FoPLs were sometimes interpreted incorrectly even when their constituent symbols were identified correctly. For instance, although the majority of participants intuited that the colour red indicated danger, a few participants did not interpret the red colouring around a symbol to be indicative of unhealthily high quantity, and instead assumed that the bright colour was there just to draw attention to the food component. Some others opined that the red colouring indicated that the packaged food contained eggs or non-vegetarian ingredients, in line with their experience of seeing FSSAI-mandated coloured dots on food packages, green on packages of foods containing only vegetarian ingredients, and brown on packages of foods containing non-vegetarian ingredients. Although the majority of participants understood that red was meant to indicate danger to health, and green indicated healthy levels, some participants emphasized that colours alone could not be relied upon to convey (un)healthiness of food components. Participants recommended the combination of text, symbols and colours to get the information across reliably. Similarly, the interpretation of symbols was mixed. Some participants interpreted the symbols for salt as flour or sugar, and the symbol of drops of fat as milk, water, or eggs.

*[FGD 5] In our India, we have so many languages. If someone doesn’t know Hindi or English, he can see the colour and get the message.*

*[FGD 5] For someone who cannot read, a black letter is no different from a water-buffalo [“Kaala akshar bhains barabar”]* [Popular saying in India]

*[FGD 2] Respondent 1: Why leave things to ambiguity when we have the language? The icons can be reduced in size but the words in English and the local language are essential.*

*Respondent 2: If in addition to the information in English, there is a bar code or QR code on every pack, and scanning it will give the person information in the local language, it would be very useful to everyone in the country.*

*[FGD 5] Is this about water, or about some other liquid? It is not clear* [just looking at the drops]… *You cannot make out if this* [spoonful of powder] *is sugar or salt.*

### 3.7 Information desired on food packages, and information expected to be used

All the participants expressed a desire for detailed information. Participants wanted details on food components, their absolute amounts in the packaged food, the amounts recommended by health authorities, and the percentages of those amounts provided by each serving of the packaged food presented on each FoPL. They wanted specific information on products’ implications for health conditions such as high blood pressure, diabetes mellitus, and chronic kidney disease. Participants also wanted detailed information on many more nutrients than those appearing on the FoPLs displayed in the FGDs, for instance, calcium, vitamins, protein, and carbohydrate. However, considering that these details were already present on the nutrition panel on the back of food packages, some groups concluded that what was vital was a summary rating or warning of unhealthy levels of certain food components presented on the front of the pack, and consumers could examine the back of the pack for more detailed information.

Participants also conceded that all the supporting information on nutrition and health could not practically be placed on labels on food packages, and needed a foundation of nutrition literacy to be useful and efficient.

*[FGD 8] Just putting grams on a label and not percentages leaves us nothing to use to calculate and compare. Of course, all the information on nutrition cannot be imparted on a label.*

Provision of comprehensive information on FoPLs was seen as a sign of transparency on the part of the manufacturer, and a way to enhance the trustworthiness of the brand. Considering the highly technical nature of the information desired on food packs, some of the participants clarified that they wanted it not for themselves to review, but for the information of, and advice from, their associates who could read and make sense of it.

Along these lines, participants appreciated interpretive FoPLs over those that merely provided information on discrete food components, particularly for packaged food for children. For instance, labels such as ‘high’, ‘medium’, and ‘low’ in addition to amounts and percentages of various nutrients were perceived as more helpful to interpret the FoPLs. Despite many stating a preference for detailed information, in most cases it was apparent that the participants relied on the more interpretive components, such as colours and rating, of the label to make assessments of the healthiness of foods. However, interpretive signalling alone in the absence of supporting information led to some confusion in viewers. For instance, the grading ‘D’ on a Nutri-Score FoPL, in the absence of any text on the components of the pack, led some participants to conclude that the food was high in vitamin D, rather than that it had received a low rating on healthiness. Also, some participants noted that a summary score was insufficient to narrow down on the particular nutrients of concern to the consumer, and would therefore not be as helpful in making purchase decisions as more detailed information on each nutrient. Thus a combination of nutrition facts, in addition to an interpretive, summary element was the most desired information on food packages. Overall, there was no clear preference between summary scores and nutrition facts. Participants wanted both so that they could make quick decisions (aided by summary scores), and more considered decisions about specific nutrients (guided by nutrition facts), the latter often with others’ help.

*[FGD 10] They should write ‘high’ and ‘low’ because sometimes we also can’t understand while choosing a product.*

*[FGD 4] How much fat and salt a person should take, people should be aware of that. If it is there, then only they can check… There should be a list of items and quantities like how much we should take and what we should not take… Also how much oil is good for us.*

*[FGD 2] If there were some graphical information that made it easy to make a decision on purchasing the food, it would better than just saying there are so many grams of this. This doesn’t make much sense to us because we are not in the field. But if we were to have a bar graph that told us how healthy a food was, that would be useful. Or if something were good for the heart, I’d like to see a heart symbol with two tick marks, as distinct from one tick mark.*

*[FGD 8] A grade or statement that a food is unhealthy is not useful for everyone. I may be trying to lose weight and so watching my fat intake. Someone else may be diabetic and so controlling sugar intake. How can we know from just a grade which is high in this particular food?*

*[FGD 8] It would be easy to set standards for children using such labels. No food that has a grade of anything lower than this, say C, will be bought. But other than that, if you are looking for information, such labels won’t do the job.*

Participants raised concerns of equity and personal responsibility in providing and receiving nutrition and health information. Across all groups, participants were sceptical of the accuracy and forthrightness of manufacturers in providing information on the nutritional value of their products. Notwithstanding this scepticism, across all FGDs there was unanimous support for mandating nutritional information, approved by health authorities as distinct from health claims made by the manufacturers, on food packages, through FoPLs that provided details on the major components of the food product, and particularly guided consumers on the (un)healthiness of the ingredients.

*[FGD 7] I think health, or being healthy, unfortunately has become a very... I don’t know how to say but... a very exclusionary process, in a way that I guess there are a very certain class of people who sort of understand how to access this information. I mean I really wonder how people, let’s say some little lesser socioeconomic background would access this information, or have the ability to access this kind of information. I think it should not be that way because people from across classes would want to be healthy. This information needs to be made very accessible… standardising it, colour-coding, something which is easily understandable, and other than, you know, using terminology and using numbers, percentage, because that is complicated.*

*[FGD 7] Not everything can be written on the packaging so I guess that kind of awareness…We will have to be more aware. We also have to educate ourselves. I don’t think that brands can write everything on a label to make it legible, but what is written can be less cryptic.*

*[FGD 9] We go by what is written on the pack. We have no real way of knowing what is actually in the food product. It is made in a company* [factory]*. We don’t know how. We just see what is written on the pack.*

### 3.8 Ranking of presented FoPLs

At the conclusion of each FGD, all the FoPL variations discussed in the course of the FGD were displayed one after another and votes solicited for or against them through participants’ calling out preferences. Five to six FoPLs were shortlisted this way in the first step, and the exercise repeated to arrive at the three top-ranked FoPLs in each FGD. From the ranking of the FoPLs presented at each FGD, the ones that were most acceptable and attractive to participants, across all the FGDs, were the HSR, MTL, FSSAI Variations 6 and 5, the Israeli warning label, and Nutri-Score, in that order. The survey that we conducted to assess objective understanding and influence on decision-making on purchases was informed by this ranking, and used 2-colour and 3-colour versions of the MTL, a colour version of the HSR, a smiley face version of the Nutri-Score, and a red Warning Label [19].

## 4. Discussion

The focus group discussions conducted among adult grocery shoppers to understand consumers’ packaged food purchasing patterns, preferences, and assessment of the (un)healthiness of foods revealed numerous influences of nutrition and health awareness, affordability, and information available in general and on food packages in particular, in decisions related to packaged food purchases. Furthermore, this study provided an understanding of the nutrition literacy, and socioeconomic and commercial context for the introduction of an FoPL by the Government of India. The current and anticipated utilisation of information presented on FoPLs, and the conditions of information access, trust, and ambiguity that need to be addressed before and alongside the implementation of an FoPL in India were explored.

For optimum effectiveness and responsiveness to context, the implementation of an FoPL needs to be preceded by intensive research to ascertain nutritional literacy, comprehensibility, and acceptability of the proposed FoPL, and the particular health and nutritional needs and background of the population [22–24]. It needs to be aligned with, and support existing measures including national food-based dietary guidelines to achieve national health and nutrition goals and promote the well-being of the population [25–28]. It also needs to be unified for a country/region, developed with inputs from researchers, industry representatives, public health activists, and consumer representatives, to be implemented and used in a standard manner for the entire population [29–31]. Our findings, and the recommendations for policy, research, and practice derived from them, respond to many of these concerns. They contribute to the knowledge on consumer perspectives on packaged foods, influences on decision-making related to the purchasing of packaged foods, and the utilisation and anticipated utility of various formats and elements of FoPLs.

Purchases were guided and influenced by taste, affordability, and perceived quality as expressed in the brand, packaging, and advertising of packaged food products. The healthiness of food products did not feature prominently in the purchasing decisions of relatively healthy low and middle-income participants. However, in the event of a diagnosis or family history of a disease condition, consumers tended to review the nutritional content of packaged food to avoid proscribed levels of certain components. High-income participants, and those with special needs owing to preferred diets and health conditions, in contrast, reported that healthiness and composition of food products were important considerations in purchasing.

Participants welcomed details on nutritional components of packaged food, in common, comprehensible, accessible terminology, pointing out and demonstrating gaps in understanding when highly technical, albeit accurate, terms were presented [32–34]. Several participants in the FGDs paid greater attention to the pictures of foods on the pack and made decisions about nutritional content and (un)healthiness based on the pictures on the packs rather than the information in the FoPLs, underscoring the need for emphasis on nutritional facts, and clear guidance on the distinction between advertising and presentation of objective information. Participants showed a strong desire for textual information in the local language in addition to information in English and Hindi. In view of the impracticality of repeating information in numerous languages on every small pack of food, manufacturers could consider placing a sticker with FoPL information in the local language on each pack, for sale in different regions.

Pande et al. [18] observed limited use of nutrition information in making food choices and decisions on purchasing among consumers in India, due to low comprehensibility of text- intensive nutrient information on the back of product packs, compared to recognition and easy comprehensibility of symbols, such as the green dot to indicate vegetarian food. The researchers proposed that a symbol-based FoPL could have better uptake and recall value and acceptability among the diverse Indian population [18]. The present study confirmed the modest level of utilisation of textual information on nutritional components, but also highlighted the confusion possible in using symbols on FoPLs without adequate verbal information to support them. Ghosh and coworkers [35] found in a large survey across India that summary FoPLs, specifically HSR and WL, were easier to identify and utilise. The findings of the present study, in particular the discussions on utility of nutrition facts and summary guidance on FoPLs and the voting on the attractiveness and acceptability of FoPLs, support this view. Our study delved deeper into the nutrition literacy (and gaps therein) and the influences on decision-making related to purchases of packaged food, and yielded several pointers for the optimisation of the design, content, and channels of nutrition information on food packaging. Bhattacharya and colleagues [36] from an online survey among literate, middle to high income consumers (mostly male), in 14 states across India, found the WL the most popular FoPL format. Our study, which elicited perspectives on FoPLs from consumers from a much wider range of socioeconomic strata, with an equal representation of men and women, to understand gender differences in purchases of packaged food, found that the WL was considered useful and attractive, but not rated as highly as several other interpretive labels.

Studies in Belgium [37], Spain [38], and Germany [39] concluded that Nutri-Score performed comparatively well in informing consumers of the nutritional quality of foods. It is important to note that the participants in these studies were all literate, internet users, and that the colours and symbols used in the FoPLs were well understood by them. Our study did not point to similar high performance or utility of Nutri-Score, suggesting rather that in the absence of an orientation to the symbols used and supporting text, non-text FoPLs may not be understood accurately, and their use may be particularly difficult in populations with low to no literacy. In our study, in the absence of clear instructions in text, and low literacy, consumers defaulted to prior experiences in using the colours and symbols on the FoPL. In this situation, it is vital that the symbology and colour-coding used are consistent with those used in prevalent campaigns, e.g., traffic lights, energy-efficiency grading, child growth charts.

Some participants expressed scepticism, and even distrust, of the veracity and credibility of information on food packages. This distrust may be largely unfounded as a 2024 study in Chennai, India, finding over 80 percent compliance of nutritional claims with food package contents, demonstrated [40]. Anecdotes of adulteration, mislabelling, and vested interests in presenting and hiding various bits of information, were related in the FGDs. Achieving the hoped for impact from implementation of an FoPL in India will need considerable groundwork in orientation of the general population to health and nutrition information, confidence-building in the regulation of the composition and safety of foods in the market [41–44], and credibility of the information presented on packs of food products. To this end and to build consumer trust in FoPLs [45,46], the general population would also be benefited by orientation to FSSAI as the accreditation authority to certify food for safe consumption.

Participants from various income and education groups reported using the internet for information and guidance on food purchases. This calls for regular monitoring and the correcting of erroneous information circulated on the internet, in tandem with the provision of accurate and concise information for the use of consumers. In view of the all-pervasiveness of mobile phones in India, it would also be expedient to develop and disseminate apps and short audiovisual material to guide decisions on healthy food purchasing.

The aggregate ranking of the study FoPLs on attractiveness and perceived utility demonstrated the participants’ preference for colourful, informative, interpretive FoPLs [47,48], easily viewed and understood, generally with text and symbols to guide consumers on making healthy purchases and avoiding unhealthy purchases. The lack of clarity on healthy foods, daily requirements, risks of deficiency and excess of particular components of food, technical terminology on nutrition [34,49–51], and non-textual information on packaged food underscores the imperative for a comprehensive, consistent, and wide-reaching nutrition literacy enhancement effort.

Many participants expressed their desire to receive information on the healthiness of foods on the packages themselves, but many others emphasised the need for greater basic awareness of nutrition and health to enhance their information and autonomy to make healthy decisions about food purchases and consumption. Low-income participants, in urban slums and rural areas, many of them with low to no educational attainment, reported their children’s input into reading and conveying information on packaged food, highlighting children as a channel for health information into the community. Low nutrition literacy was far more wide-ranging than low educational attainment in our study participants, highlighting the need for focused awareness-raising on nutrition, and avoiding the assumption that standard education would automatically improve nutrition literacy. Regardless of the particulars of the FoPL decided on for nationwide implementation, the government needs to promote health and nutrition literacy, and institute policy and provision supportive to the uptake of fresh, safe, and healthy foods [52,53], to facilitate better population health, including the optimal use of the FoPL implemented. A robust nutrition awareness campaign needs to be implemented before and alongside the introduction of an FoPL in India. This awareness raising can be operationalised through formal education (in schools and colleges) and through mass and folk media.

Participants felt that FoPLs should be uniform across categories of packaged food, to facilitate easy comparison and decision-making, and adhere to unambiguous standards of measurement, i.e., weights and volumes rather than servings. All participants were keen on legislation to make informative and interpretive FoPLs mandatory on packaged foods. This is in line with the observation that keeping FoP labelling voluntary discourages widespread adoption of FoPLs [25,54,55], and resistance from the food industry leads to implementation delays [55–57].

The study findings also have implications for food industry actors. Participants consistently associated clear, government-endorsed nutrition information with greater trustworthiness, while expressing scepticism towards manufacturer-led health claims. Food manufacturers could aid consumer understanding by presenting nutrient information using consistent terminology, larger and more legible fonts, high-contrast colours, and standardised reference units (e.g., per 100 g/ml). In addition, reformulation to reduce nutrients of concern, including salt, sugar, and saturated fat, would enable manufacturers to achieve more favourable FoPL outcomes, and greater acceptability among consumers.

### 4.1 Strengths and limitations of the present study

The geographic and socioeconomic variation covered in the FGDs makes these findings applicable to a wide range of Indian grocery shoppers. The topics explored in the FGDs provide a nuanced understanding of food purchase patterns, influences on decision-making, beliefs underlying dietary behaviours, consumers’ needs and preferences, and some pointers towards effective content and channels of messaging to transmit nutrition information. The range of FoPL variations used stimulated discussion on a wide variety of information pathways (e.g., images, text, symbols, colours, graphics) and the pros and cons in their use.

Our study could be conducted in only two zones of the country, viz. North and South. A geographically more dispersed study could have thrown up more findings, and clarified regional differences and similarities to a greater extent. We conducted FGDs only with adults who were regular grocery shoppers for their households. Discussions with persons of other age groups, e.g., children, adolescents, and elderly persons, could have shed light on their beliefs, preferences, and influences on the purchase of foods in their households. As this study relied entirely on what participants chose to report about their own experiences, the data that we collected may have been partial or biased, in contrast to non-participant observation of actual interactions in households and actual purchases, which were beyond the scope of this project.

### 4.2 Policy implications

This study yields numerous insights into the needs of, prevailing gaps in, and potential enablers and avenues for the enhancement of nutrition literacy and information provision for healthier food choices in the Indian population. Policy recommendations for the design, content, and preparation and implementation of an effective FoPL in India may be encapsulated as follows:

i Conduct a robust nationwide nutrition and health literacy campaign, using formal education and community engagement approaches.
ii Develop an interpretive FoPL that is viewer-friendly, informative, in line with prevalent signage in the country, and widely comprehensible through the use of colloquial terminology in English, Hindi, and as far as possible in the local languages as well. Further, keep the FoPLs visually interesting though occasional modifications to prevent their getting neglected after the initial novelty wears off.
iii Familiarise consumers with nutrition accreditation authorities (e.g., FSSAI), and mandate FoPLs, with basic guidance on their utilisation, on all packaged food.

The Government of India has, through its Food Safety and Standards (Labelling & Display) Amendment Regulations, 2022, published in the Official Gazette, draft notified the Indian Nutrition Rating (INR) FoPL with a pictorial display format, adapted from the HSR [58]. The INR gives packaged food a rating from ½ to 5 stars, using red colouring on the background strip. The FoPL is to be displayed in close proximity to the brand name on the front of the pack. The FoPL also provides for the presentation of additional interpretive information on the nutritional factors deemed to increase health risks in the population. This is yet to be finalised and implemented. The INR proposed is supported by several of our study’s findings and recommendations. Future research to evaluate the impact of the FoPL implemented, and design revisions to enhance the ability of the FoPL and the consumers to use it to guide healthy food choices, is warranted.

## Supporting information

S1 File Focus group discussion guide. (DOCX)

S2 File Variations of the draft FSSAI FoPL presented in the preliminary FGDs. (DOCX)

S3 File Modified FoPLs used in the FGDs. (DOCX)
