# Supplementary material for: Designing front-of-pack labels for packaged food in low-literacy contexts: Consumer perspectives from India
Source: PLOS Glob Public Health. 2026 Aug 25;6(8):e0007118. doi: 10.1371/journal.pgph.0007118 (PMC13505920; doi:10.1371/journal.pgph.0007118)
Supplement: Supplementary file 1 [file pgph.0007118.s001.docx]

*S1. Focus group discussion guide*

| Topics | Subtopics | Prompts / questions |
| --- | --- | --- |
| Food purchasing patterns (*icebreakers & orientation*) | Frequency of purchasing  Site of purchasing  Selection criteria | How often do you purchase groceries?  Where do you purchase groceries?  How do you decide which food products to buy? What factors do you consider before you buy a food product? [*Prompt for unhealthiness: Is the unhealthiness of the food, or the impact that the food will have on your health, a factor that you consider in choosing a food product?*] |
| Nutrition information | Indicators of healthiness of foods  Nutritionally ambiguous products  Content and format of information desired on packs | How do you tell if a food is healthy or not? [*Check for mention of on-pack nutrition information*] Do you face any difficulty in understanding which food products are unhealthy and which healthy?  Which food items do you face such difficulties with?  Which kinds of foods that you buy regularly are most difficult to assess for unhealthiness? [*to inform the survey in Phase II*]  What types of nutrition information do you desire on the pack? Is there any particular information on nutrition that would be helpful for you? *[Prompt for particular health conditions that may require special attention to nutrition, e.g. high blood pressure, diabetes mellitus, kidney disease, gout, thyroid disorders, overweight/obesity.]*  Please tell us what you understand by these words: energy/calories, total sugar/added sugar, salt/sodium, total fat/saturated fat?  Are you aware of the nutrition facts on the back of packs? Do you use them?  Have you seen any nutrition labelling on the front of packs? Do you use it? [Sensitise participants to the concept of FoPLs.]  What other types of nutrition information would you like on the front of packs?  How should this information be provided:  Would colours be helpful? Which colours would be good?  Which languages would be useful?  Which shapes and symbols would be good? |
| Perceptions of draft FSSAI FoPL  Perceptions of variants of draft FSSAI FoPL  Perceptions of various FoPLs | Acceptability  Perceived effectiveness  Noticeability  Legibility  Informativeness  Understanding  Speed of product comparisons  Trustworthiness  Extent to which FoPLs will persuade participants not to buy unhealthy products and to buy healthier products | What is your opinion of this FoPL? *Prompt for the following if they do not emerge from discussions:*  *Is it noticeable? [Prompt for size, placement.]*  *Is it clear?*  *Is it informative?*  *Is it easy to understand?*  *Can you compare foods using this FoPL?*  *Do you need such information for all foods?*  *Do you need such information for specific foods? If yes, what kinds of food?*  *Would you find such information useful in making decisions about unhealthy foods and healthy foods?*  *How would you use the information in the FoPL? Would you avoid buying unhealthy foods? Would you buy healthier foods*?  What would you like to see changed in this FoPL? |
| Comparison of acceptability and effectiveness across all FoPLs | Rating of FoPLs | You have seen and discussed many different FoPLs.  Which did you find most useful?  Which did you find less useful?  What would you like to see on FoPLs in the future? |
| Circumstances/contexts of use of FoPLs |  | When would you use information on FoPLs?  first versus regular purchase  shopping for self versus shopping for others  purchasing healthy versus unhealthy food categories |
| Views on mandatory versus voluntary front-of-pack labelling |  | Do you feel that FoPLs should be made compulsory, or should manufacturers be able to decide whether or not to have FoPLs on their products? |
