# Supplementary material for: Designing front-of-pack labels for packaged food in low-literacy contexts: Consumer perspectives from India
Source: PLOS Glob Public Health. 2026 Aug 25;6(8):e0007118. doi: 10.1371/journal.pgph.0007118 (PMC13505920; doi:10.1371/journal.pgph.0007118)
Supplement: Supplementary file 2 [file pgph.0007118.s002.docx]

*S2. Variations of the draft FSSAI FoPL presented in the preliminary FGDs*

Draft FoPL developed by FSSAI:

- per serve
- calories, saturated fat, trans fat, added sugar, sodium
- units: kcal, g, mg
- red crescent to indicate high amount

- per serve
- calories, saturated fat, trans fat, added sugar, sodium
- units: kcal, g, mg
- red crescent to indicate high amount
- Hindi translations of food components
- per serve
- calories, saturated fat, trans fat, added sugar, sodium
- units: kcal, g, mg
- Red, amber, and green crescents to indicate amounts
- per serve
- energy, saturated fat, salt, total sugar
- units: kcal, g
- red box to indicate high amount
- per serve
- energy, saturated fat, salt, total sugar
- units: kcal, g
- red crescent to indicate high amount
