# Supplementary material for: Designing front-of-pack labels for packaged food in low-literacy contexts: Consumer perspectives from India
Source: PLOS Glob Public Health. 2026 Aug 25;6(8):e0007118. doi: 10.1371/journal.pgph.0007118 (PMC13505920; doi:10.1371/journal.pgph.0007118)
Supplement: Supplementary file 3 [file pgph.0007118.s003.docx]

*S3.**Modified FoPLs used in the FGDs*

FSSAI FoPL with outside crescent area coloured

FSSAI FoPL with icons

FSSAI FoPL without percentages

FSSAI FoPL with 2 colours

FSSAI FoPL with per 30 g

FSSAI FoPL with addition of Hindi language

FSSAI Draft FoPL

Guideline Daily Amount

FSSAI FoPL with icons

Chilean Warning Label

Israeli Warning Label

Nutri-score

Health Star Rating

Guideline Daily Amount

Multiple Traffic Lights

Chilean Warning Label

Israeli Warning Label

Multiple Traffic Lights

Nutri-score

Health Star Rating
